# Harmonized global soil carbon and respiration datasets with derived turnover time and temperature sensitivity

**DOI:** 10.1038/s41597-025-06254-4

**Published:** 2025-12-11

**Authors:** Shoji Hashimoto, Akihiko Ito, Kazuya Nishina

**Affiliations:** 1 Department of Forest Soils, Forestry and Forest Products Research Institute, Tsukuba, Ibaraki 305-8687 Japan; 2 Graduate School of Agricultural and Life Sciences, The University of Tokyo, Bunkyo-ku, Tokyo 113-8657 Japan; 3 Earth System Division, National Institute for Environmental Studies, Tsukuba, Ibaraki 305-8506 Japan

**Keywords:** Carbon cycle, Carbon cycle

## Abstract

Soil carbon stocks and their release to the atmosphere are key processes for accurately predicting future climate–terrestrial carbon feedbacks. However, data-driven estimates of these variables exhibit substantial variability across studies. In this work, we compiled all publicly available global maps of soil carbon stock and heterotrophic respiration derived from observational data, converted them into NetCDF format, and standardized them to a 0.5-degree spatial resolution. We calculated the mean, maximum, and minimum values for each grid cell to generate harmonized global maps of both variables. From these harmonized datasets, we further derived estimates of soil carbon turnover time and its temperature sensitivity. The resulting products provide valuable benchmarks for the development, evaluation, and constraint of terrestrial carbon cycle models in Earth system science.

## Background & Summary

Accurately predicting future climate change requires a well-constrained terrestrial carbon cycle^1,2^. Soils represent the largest reservoir of organic carbon in terrestrial ecosystems, and the release of CO₂ from soils to the atmosphere constitutes the second-largest carbon flux between the biosphere and the atmosphere^3^. Consequently, soil carbon (C) stocks and soil CO₂ efflux—particularly heterotrophic respiration (*R*_H_) associated with the decomposition of soil organic carbon—are key processes^4–6^. Data-driven estimates of both soil C stocks and fluxes are critical for constraining terrestrial carbon cycle models^4,7,8^.

Over the past few decades, although not as extensively studied as net primary productivity^9^, substantial efforts have been made to quantify soil C stocks and *R*_H_^10–16^. These efforts have yielded various global datasets, often based on thousands of observational data points and upscaling techniques such as machine learning. Nevertheless, considerable uncertainty persists in global estimates of soil C and respiration—even among data-driven products^17,18^.

In practice, a major obstacle to the effective use of these datasets lies not only in the variability among estimates but also in inconsistencies in data formats and spatial resolution (e.g., NetCDF [Network Common Data Format], GeoTIFF [Georeferenced Tagged Image File Format], and MATLAB files; ranging from 30 arc-seconds to 0.5 degrees). These issues hinder the seamless integration and comparative use of global soil C and respiration data.

To address this challenge, we compiled all publicly available global maps of soil C stocks and *R*_H_, standardized them into a common NetCDF format at a 0.5-degree spatial resolution, and generated harmonized maps representing the mean, maximum, and minimum values at each grid cell. From these harmonized datasets, we also derived estimates of soil C turnover time and temperature sensitivity, which are fundamental metrics in terrestrial soil carbon cycle. Together, these products provide a valuable foundation for improving, validating, and constraining terrestrial carbon cycle models in Earth system science.

## Methods

We collected all available global soil C maps and *R*_H_ maps derived from data-driven estimates, sourced from repositories and the supplementary materials of previous studies (Table 1). All spatial datasets were converted into NetCDF format and processed using CDO (version 2.1.1; Climate Data Operators, https://code.mpimet.mpg.de/projects/cdo/), QGIS (version 3.26; https://www.qgis.org/en/site/), NetCDF-C (version 4.9.0), GDAL (version 3.6.2), and R (version 4.2.2).

**Table 1:** List of soil carbon and heterotrophic respiration datasets used in this study.

Dataset	Repository/References (Dataset name)	Depth
Global soil C	Global soil data task 2000 (IGBP-DIS)^24^	0–100
	Shangguan *et al*. 2014 (GSDE)^22,28^	0–100, 0–30*
	Batjes 2016 (WISE30sec)^27,29^	0–100, 0–30
	Sanderman *et al*. 2017 (Soil-Carbon-Debt)^26,30^	0–100, 0–30
	Soilgrids team and Hengl *et al*. 2017 (SoilGrids)^14,46^	0–30**
	Hengl and Wheeler 2018 (LandGIS)^25^	0–100, 0–30
	FAO 2022 (GSOC)^15^	0–30
	FAO 2023 (HWSD2)^23^	0–100, 0–30
Circumpolar soil C	Hugelius *et al*. 2013 (NCSCD)^31–33^	0–100, 0–30
Global *R*_H_	Hashimoto *et al*.^10,37^	—
	Warner *et al*. 2019 (Bond-Lamberty equation based)^11,38^	—
	Warner *et al*. 2019 (Subke equation based)^11,38^	—
	Tang *et al*.^12,39^	—
	Lu *et al*.^35,40^	—
	Stell *et al*.^34,41^	—
	Yao *et al*.^36,42^	—
	He *et al*.^13,43^	—

^*^The vertical depth intervals did not exactly match 100 cm and 30 cm. Therefore, weighted means were calculated for the 0–100 cm and 0–30 cm depths. **Only the soil C stock data for the 0–30 cm depth is officially provided in the repository.

**Fig. 1:**
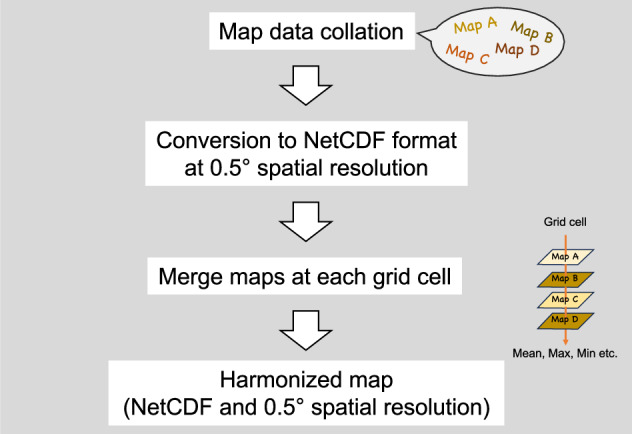
Schematic of the workflow for dataset harmonization. Spatial map data of soil carbon and heterotrophic respiration were converted to NetCDF format and then harmonized to a common spatial resolution. For *R*_H_ datasets with multi-year values, the temporal mean was calculated prior to spatial aggregation. NA values were assigned to grid cells with fewer than three soil carbon estimates or fewer than four heterotrophic respiration estimates.

Because the maps had varying spatial resolutions (from 0.0083° to 0.5°), we harmonized all datasets to a common resolution of 0.5° (approximately 50 km resolution at the equator) (Fig. 1). For soil C datasets with finer resolution, we calculated the area-weighted mean values at the 0.5° grid scale. Grid cell areas were computed using the gridarea operator in CDO. For *R*_H_ datasets with multi-year values, the temporal mean was calculated prior to spatial aggregation.

We merged the processed maps by computing the mean, maximum, and minimum values at each grid cell, resulting in harmonized global maps of soil C (for the top 100 cm and 30 cm) and *R*_H_, at 0.5° resolution (Fig. 1). Differences in the boundaries of the original maps and spatial conversions occasionally led to grid cells with too few estimates, mainly along the edges of water bodies, deserts, and ice barrens. To ensure consistency, we applied thresholds for the minimum number of estimates required: grid cells with fewer than three soil C estimates or fewer than four *R*_H_ estimates were assigned NA values (Fig. 1). Land and water grid cells were automatically distinguished by merging multiple datasets and identifying cells with sufficient soil C/*R*_H_ estimates.

The *R*_H_-based soil C turnover time (years), denoted *τ*, was calculated under the assumption of a quasi-equilibrium state using the equation:

$$\tau =\frac{{C}_{{\rm{S}}}}{{R}_{{\rm{H}}}}$$

where *C*_S_ is soil C stock and *R*_H_ is the heterotrophic respiration rate. The uncertainty range of *τ* was calculated for each grid cell using:

$${\tau }_{\max }=\frac{{C}_{{\rm{S}}}^{+}}{{R}_{{\rm{H}}}^{-}},\,{\tau }_{\min }=\frac{{C}_{{\rm{S}}}^{-}}{{R}_{{\rm{H}}}^{+}},$$

where *C*_S_^+^ and *C*_S_^−^ are the maximum and minimum soil C values, and *R*_H_^+^ and *R*_H_^−^ are the maximum and minimum *R*_H_ values, respectively.

To calculate the temperature sensitivity of decomposition (*Q*_10_)—the factor by which decomposition rates increase with a 10 °C rise in temperature, we followed the method of Koven *et al*.^19^. Using the decomposition rate constant *k*, *Q*_10_ is expressed as:

$${Q}_{10}={\left(\frac{k(T)}{{k(T}_{{ref}})}\right)}^{\frac{10}{(T-{T}_{{ref}})}}$$

Given that *k* = 1/*τ*, *Q*_10_ can be written as:

$${Q}_{10}={\left(\frac{{\tau (T}_{{ref}})}{\tau (T)}\right)}^{\frac{10}{(T-{T}_{{ref}})}}$$

Taking the logarithm of both sides yields:

$${\log (Q}_{10})=\log {\left(\frac{{\tau (T}_{{ref}})}{\tau \left(T\right)}\right)}^{\frac{10}{\left(T-{T}_{{ref}}\right)}}$$

$${\log (Q}_{10})=-10\frac{\log \left(\tau \left(T\right)\right)-\log ({\tau (T}_{{ref}}))}{(T-{T}_{{ref}})}$$

From this, the temperature sensitivity can also be expressed as:

$${Q}_{10}={10}^{\left(-10\frac{d\log (\tau )}{{dT}}\right)}$$

We applied a quadratic fit to the relationship between annual mean temperature *T* and log(*τ*)

$$\log \left(\tau \right)=a{T}^{2}+{bT}+c$$

yielding the final expression for *Q*_10_:

$${Q}_{10}={10}^{-10\left(2{aT}+b\right)}.$$

The uncertainty of *Q*_10_ (maximum and minimum values) was derived using *τ*_max_ and *τ*_min_, respectively. All statistical analyses, including curve fitting, were conducted in R (version 4.2.2)^20^.

## Data Records

All harmonized datasets are available at the Zenodo repository (10.5281/zenodo.15110782)^21^.

The collated datasets are listed in Table 1. We found six global soil C maps for 0–100 cm, seven for 0–30 cm^15,22–30^, and one regional soil C map for north circular regions for both depths^31–33^, and eight *R*_H_ maps^10–13,34–43^.

All files are provided in NetCDF format. The soil C file includes the following variables:

- longitude, latitude
- soc: mean soil C stock (kg C m−2)
- soc_median: median soil C (kg C m−2)
- soc_n: number of estimates per grid cell
- soc_max, soc_min: maximum and minimum soil C (kg C m−2)
- soc_max_id, soc_min_id: study IDs corresponding to the maximum and minimum values
- soc_range: range of soil C values
- soc_sd: standard deviation of soil C (kg C m−2)
- soc_cv: coefficient of variation (%)

The *R*_H_ file includes:

- longitude, latitude
- rh: mean *R*_H_ (g C m−2 yr−1)
- rh_median, rh_n, rh_max, rh_min: as above
- rh_max_id, rh_min_id: study IDs for max/min
- rh_range, rh_sd, rh_cv: analogous variables for *R*_H_

The mean, maximum, and minimum values of soil C turnover time are provided as separate files. The *Q*_10_ files contain estimates derived from the mean values of soil C and *R*_H_, along with associated uncertainty values.

The harmonized dataset files available in the repository are as follows:

- harmonized-RH-hdg.nc: global soil heterotrophic respiration map
- harmonized-SOC100-hdg.nc: global soil C map for 0–100 cm
- harmonized-SOC30-hdg.nc: global soil C map for 0–30 cm
- Q10.nc: global Q10 map
- Turnover-time_max.nc: global soil C turnover time estimated using maximum soil C and minimum *R*_H_
- Turnover-time_min.nc: global soil C turnover time estimated using minimum soil C and maximum *R*_H_
- Turnover-time_mean.nc: global soil C turnover time estimated using mean soil C and *R*_H_
- Turnover-time30_mean.nc: global soil C turnover time estimated using the soil C map for 0–30 cm

## Technical Validation

**Fig. 2:**
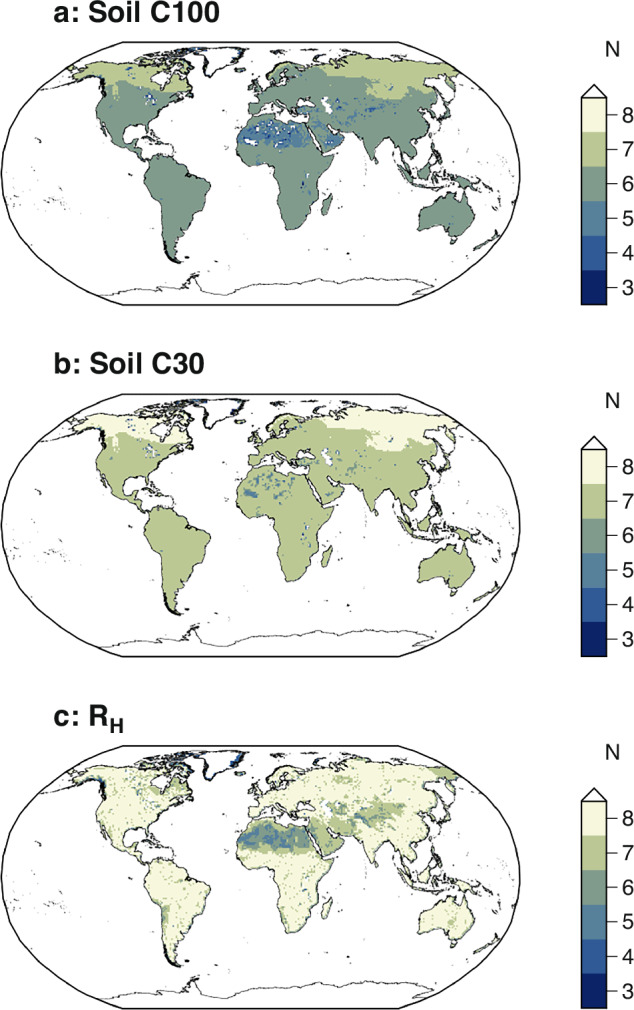
Number of input datasets per grid cell. Global maps showing the number of available soil carbon (**a,b**) and heterotrophic respiration (**c**) datasets used in the harmonization process for each 0.5° grid cell.

**Fig. 3:**
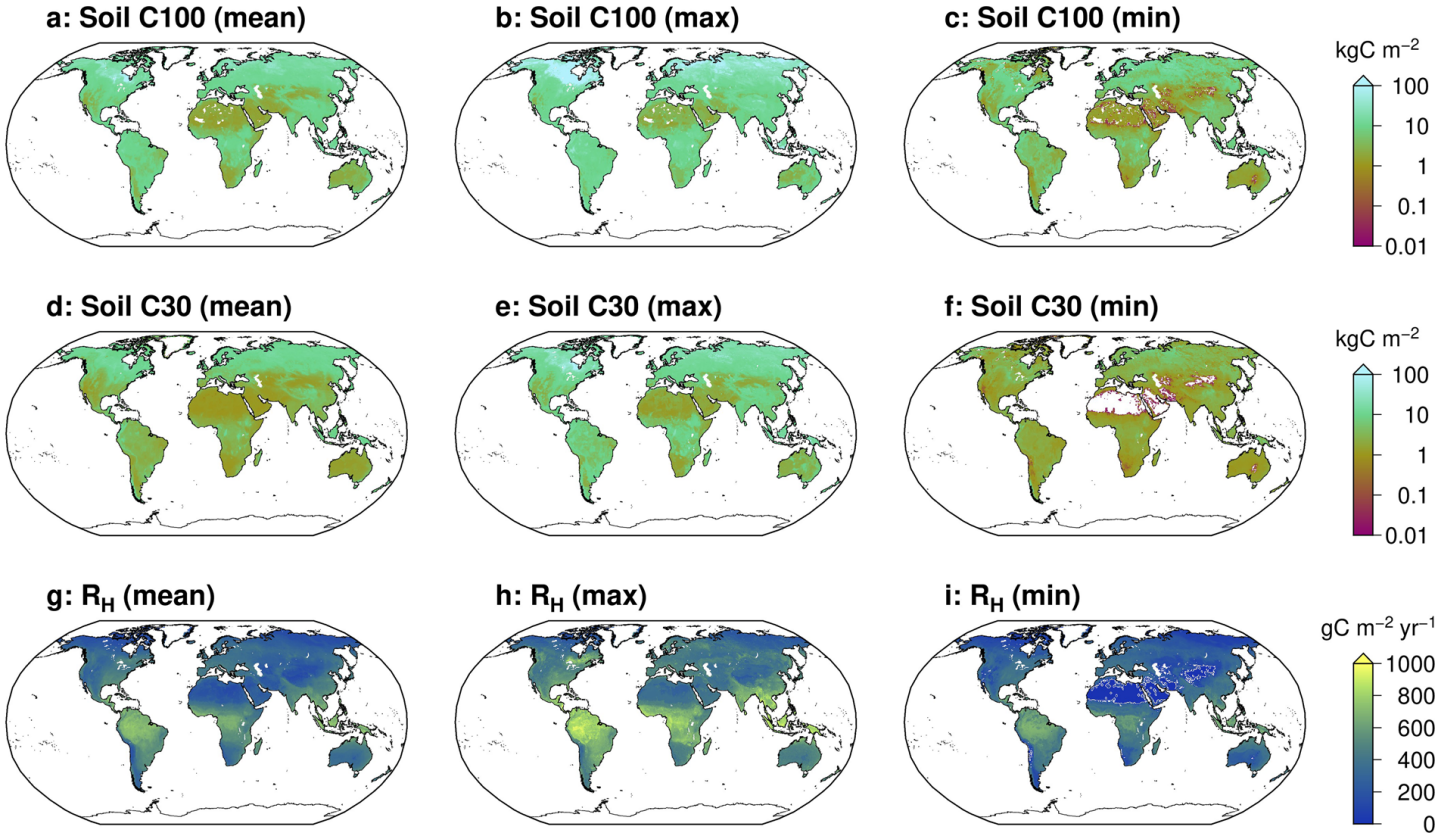
Components of the harmonized soil carbon and heterotrophic respiration datasets. Global maps showing the (**a**–**c**) soil carbon stock for the top 100 cm, (**d**–**f**) soil carbon stock for the top 30 cm, and (**g**–**i**) heterotrophic respiration. For each variable, the mean (**a,d,g**), maximum (**b,e,h**), and minimum (**c,f,i**) values across available datasets are shown at 0.5° spatial resolution.

**Fig. 4:**
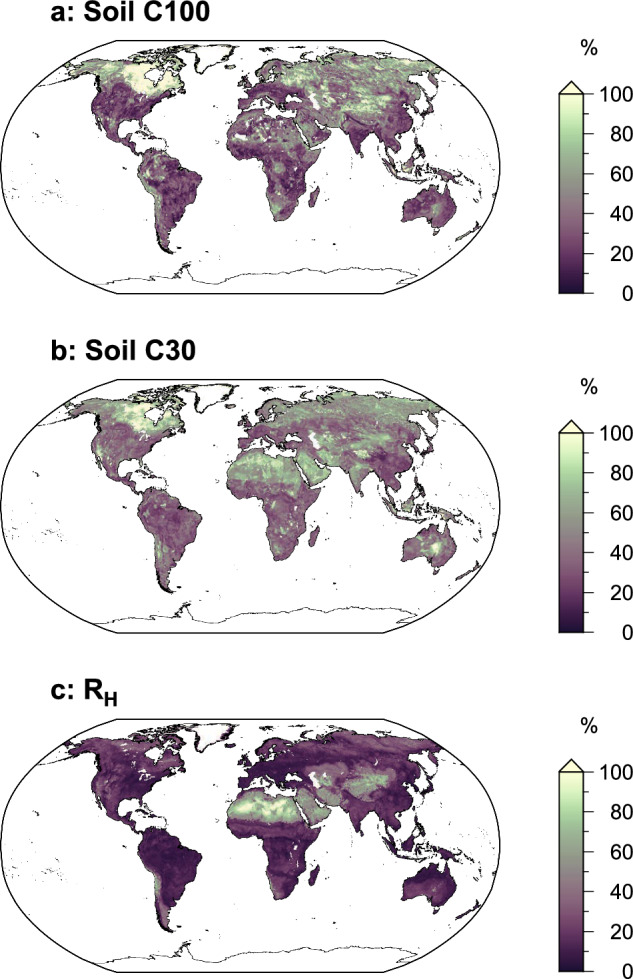
Coefficient of variation for harmonized soil carbon and heterotrophic respiration estimates. Maps show the coefficient of variation (%) for (**a**) soil carbon stock in the top 100 cm, (**b**) soil carbon stock in the top 30 cm, and (**c**) heterotrophic respiration. Higher values indicate greater variability among the underlying datasets for each grid cell.

**Fig. 5:**
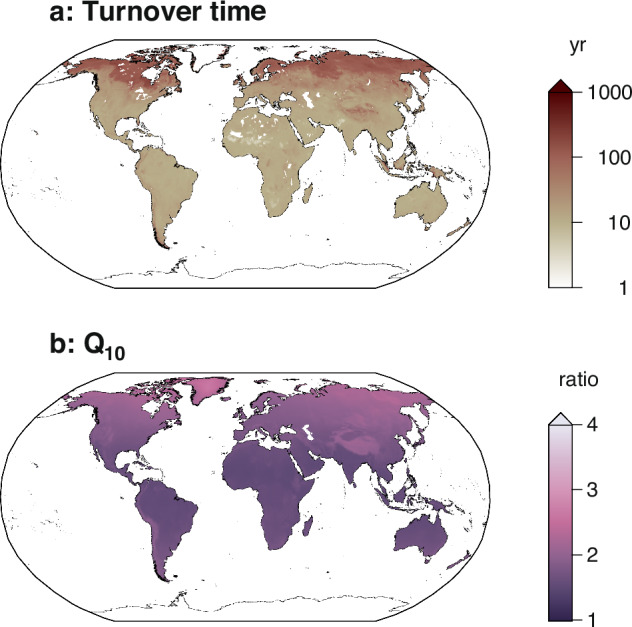
Derived soil carbon turnover time and temperature sensitivity (*Q*_10_). Maps show the estimated (**a**) soil carbon turnover time (years) for the 0–100 cm depth and (**b**) the temperature sensitivity of decomposition (*Q*_10_), calculated from the harmonized soil carbon and heterotrophic respiration datasets.

We calculated the total amounts of soil C and *R*_H_ and visualized their spatial distributions and derived turnover time and *Q*_10_ (Figs. 2–5). The global sum of mean soil C was estimated at 1855 Pg C for the 0–100 cm depth, with a maximum value of 3640 Pg C, a minimum of 933 Pg C, and a global total based on grid-cell median values of 1579 Pg C. For the 0–30 cm depth, the total mean soil C was 958 Pg C, ranging from 438 to 1845 Pg C, with a global total based on grid-cell median values of 842 Pg C. The global mean *R*_H_ was estimated at 52.8 Pg C yr−1, with a maximum of 67.1 Pg C yr−1, a minimum of 39.8 Pg C yr−1, and a global total based on grid-cell median values of 52.6 Pg C yr−1. These values are consistent with those reported in previous studies, and the spatial patterns observed in our harmonized maps broadly agree with earlier global assessments^10,11,17,34^. The derived estimates of soil carbon turnover time and temperature sensitivity (*Q*_10_) are consistent with values reported in previous studies^17,19,44,45^. These comparisons support the validity of the harmonized dataset for use in large-scale carbon cycle assessments.

## Usage Notes

The datasets are provided in NetCDF format, making them easily accessible and compatible with a wide range of tools and programming languages commonly used in earth system and environmental sciences. These include CDO, NetCDF Operators (NCO), QGIS, Python, R, ncview, and C/C++, among others. For example, users can calculate the total global soil C stock using CDO by applying a grid-area-weighted summation command. A representative command-line example is:

*cdo -output -divc,1000000000000 -fldsum -mul -selname,soc ensmean-R-soc.nc -gridarea > global-sum-output.dat*

It should be noted that the dataset contains only harmonized summary statistics, such as the mean, maximum, minimum, and range of estimates, and does not include the original individual datasets from each source study. Additionally, the total values and summary statistics of soil C and *R*_H_ may vary depending on how the data are combined with other variables (such as temperature or precipitation), since calculations are restricted to grid cells with valid data across all variables involved. It is also important to be aware that the original individual datasets carry their own uncertainties stemming from different data sources and mapping techniques, and these uncertainties are not explicitly incorporated into the harmonized datasets. Another source of uncertainty arises from the different temporal representativeness between the soil C and *R*_H_ datasets. Although it is difficult to evaluate the impact, this issue may be particularly significant in northern regions where rapid climate warming is occurring.

The harmonized soil carbon datasets are created by simply overlaying the different soil C/*R*_H_ datasets. If users wish to distinguish specific soil types such as peat or permafrost soils, they will need to overlay these datasets with additional maps representing the distribution of peatlands and permafrost.

## Data Availability

The harmonized datasets are openly available on Zenodo: 10.5281/zenodo.15110782.

## Code availability

The code used to harmonize the global soil C and *R*_H_ datasets in this study is openly available on Zenodo: 10.5281/zenodo.15110782.
